# Suboptimal monitoring of glucose levels and poor glycaemic control is associated with increased mortality and length of stay in adult inpatients with diabetes in a tertiary New Zealand hospital

**DOI:** 10.1900/RDS.2023.19.43

**Published:** 2023-06-30

**Authors:** Dave A Duggan, Lynne M Chepulis, Natasha Brown, Chris Wang, Justina E Wu, Ha Nguyen, Ryan G Paul

**Affiliations:** 1 Waikato Regional Diabetes Service, Waikato District Health Board, Hamilton, 3204, New Zealand,; 2 Waikato Medical Research Centre, University of Waikato, Hamilton, 3204, New Zealand.

**Keywords:** diabetes, glycaemic control, hospital, inpatients Māori, New Zealand mortality

## Abstract

**Objectives:**

We aimed to determine the effectiveness of glycaemic monitoring and control in the inpatient setting of a tertiary New Zealand hospital, and whether suboptimal control and monitoring may be associated with adverse outcomes in both Māori and Non-Māori diabetes patients.

**Methods:**

Clinical records including all glucose levels (n = 51,680) from inpatients ≥ 15 years old with diabetes who were admitted to Waikato Hospital for > 24 hours between 1st July 2017 to 30th June 2018 were extracted electronically from the hospital database, and the data retrospectively examined (n=3,380 patients and 4,901 admissions).

**Results:**

Overall 80.8% of diabetes inpatients had their blood glucose levels monitored. Patients experiencing ≥ 1 episode of hypoglycaemia were 1.90 times (CI: 1.37-2.64) and 1.94 times (CI: 1.51-2.49) more likely to die within 60 days and one year respectively, with an increased length of hospital stay by a mean of 3.13 weeks (CI: 2.55-3.85). Māori patients were more likely to experience ≥ 1 episode of hypoglycaemia (OR: 1.46), with a higher one-year mortality (p<0.001) as well as higher readmission rates at 30,60, 90 and 365 days than non-Māori. Blood glucose checks at least once every 24 hours were associated with shorter hospital stays (0.36 weeks) and a lower one-year mortality (Adjusted odds ratios (OR): 0.77, CI: 0.64-0.91).

**Conclusions:**

At least one episode of inpatient hypoglycaemia was associated with a statistically significant increase in 60-day and one-year mortality as well as notably longer hospital stays, with more frequent hypoglycaemia occurring in Māori patients. Significant hyperglycaemia was associated with an increased one-year mortality, higher readmission rates within one year and longer hospital stays.

## 1 Introduction

A total of 18.5% of medical and surgical beds at Waikato hospital, New Zealand in 2018 accounted for patients with diabetes [1]. Similarly, it is estimated that in the United Kingdom in 2019 approximately 20% of inpatients at any given time had a diagnosis of diabetes, with hyperglycaemia being present in 22-46% of non-critically ill patients [2]. Dysglycaemia is linked to increased morbidity, mortality and prolonged length of stay with subsequent increased health costs independent of cause of admission [3]. The estimated annual cost of type 2 diabetes in New Zealand in 2021 was 2.1 billion NZ dollars (0.67% GDP) with an expected increase in type 2 diabetes diagnoses of 70-90% in the next 20 years [4].

Whilst very tight glycaemic control may increasemortality in the critically ill [5], the benefits of treatment of hyperglycaemia are evident with lower levels of hospital complications in both medical and surgical patients [6]. International guidelines recommend treatment of glucose levels >10mmol/L in inpatients and perhaps even tighter control in select patients who are not critically ill [7]. The recommended minimum frequency of glucose monitoring levels in inpatients is at least before every meal or every 4-6 hours in those who are not eating. Patients on intravenous insulin infusions need more frequent monitoring [7]. Glycaemic monitoring is traditionally via capillary glucose levels, but the use of continuous glucose monitoring in the inpatient setting is becoming more common.

Currently there is a scarcity of published data relating to New Zealand inpatient glycaemic monitoring and control necessitating this retrospective review undertaken at a 673 bed New Zealand urban tertiary care hospital, with both an urban and rural catchment area of 25,598km^2^[1]. In the Waikato hospital catchment region in 2018, Māori, the indigenous people of New Zealand, had 5,624 people living with diabetes with 13,838 non-Māori with diabetes [1]. There was ethnic disparity in diabetes hospital admissions at the time of the study with 32.1% of the Pacific Islanders having diabetes, 24.6% of Asians, 22.9% of Māori and 15.9% of European or ‘other’ having diabetes. This data demonstrates a higher diabetes inpatient representation in Asian and Māori and under-representation of Pacific Islander population, with prevalence of known diabetes in these populations being 11.1% in Pacific Islanders, 5.7% of Asians, 5.3% of Māori and 4.7% in European or other.^1^ We aimed to assess the frequency of glucose monitoring and control in such a setting as well as whether glycaemic abnormalities were associated with increased readmission rates and mortality.

Māori have inequitable health outcomes in New Zealand, living seven years shorter than non-Māori, with reduced access to health services and higher rates of disability compared with non-Māori [8]. We therefore also aimed to assess if sub-optimal glycaemic monitoring and control is associated with adverse outcomes in Māori patients with diabetes compared with non-Māori.

## 2 Methods

Electronic data were obtained for all patients ≥ 15 years of age who were admitted to Waikato Hospital for > 24 hours between 1^st^ July 2017 to 30^th^ June 2018 with an International Classification of Diseases (ICD) code of either type 1 or type 2 diabetes. Pregnant patients were excluded from the study due to different glycaemic recommendations [9]. Patients who did not have any glucose measurements during their hospital stay were excluded from analyses on glycaemic events. The Waikato Hospital policy during the study time period was for all capillary blood glucose and ketone levels to be performed by ward staff using certified point of care testing on each ward and patient glucometers were not to be used. Any flash or continuous glucose monitoring readings were to be confirmed with capillary blood glucose measurement. The results of point of care testing were automatically uploaded to their electronic records. Glycosylated haemoglobin (HbA1c) levels and date of test were obtained from hospital and community laboratories from 1^st^ March 2017 to 30^th^ June 2018.

Data collected for analysis comprised: number of admissions in patients with diabetes irrespective of the cause of admission; number of patients; patient demographics including age, gender, ethnicity and socioeconomic status; frequency of capillary blood glucose and ketone monitoring; number and severity of hyperglycaemic and hypoglycaemic events; length of stay; critical care admissions. For the purposes of analysis, the following glycaemic ranges were used: Hypoglycaemia ≤ 3.9 mmol/L; target range blood glucose 4.0-10.0 mmol/L; mild hyperglycaemia 10.1-13.9 mmol/L; significant hyperglycaemia ≥ 14 mmol/L [10].

Characteristics and outcomes of patient admissions were compared between those having at least one episode of hypoglycaemia (≤ 3.9 mmol/L), hyperglycaemia (> 10 mmol/L) and/or significant hyperglycaemia (≥ 14 mmol/L) to those without these events; by using Chi-squared tests for categorical variables and Student’s t-tests for continuous variables. The data was analysed to provide information regarding outcomes in relation to glycaemic parameters, length of stay, mortality, readmissions, critical care admissions, socioeconomic status and ethnicity. Ethnicity statistics were prioritised for Māori as this was an ethnicity of interest in this study. The New Zealand Deprivation 2013 (NZDep2013) index score was used as a marker of socioeconomic status, with higher scores indicating greater levels of social deprivation [11].

Logistic regressions were used to examine the association between outcomes (mortality and readmission) and suboptimal monitoring of glucose levels and glycaemic control (hypoglycaemia, any hyperglycaemia, significant hyperglycaemia, and glucose checks every 24 hours at least once) adjusting for type of diabetes, ethnicity, and age. We also ran Ordinary Least Squares (OLS) regressions to investigate the impact of suboptimal monitoring of glucose levels and glycaemic control on the length of stay, adjusting for type of diabetes, ethnicity, and age. Logistic regressions were used to assess the ethnic differences in the monitoring of glucose levels and glycaemic control adjusting for age and type of diabetes. Data analyses in Tables 1 and 2 were performed in Stata 15 (StataCorp LLC, Texas, USA).

**Table 1.** Demographics, clinical characteristics and patient outcomes

Factors	Values	Admissions had at least one episode of hypoglycaemia (≤ 3.9 mmol/L)			Admissions had at least one episode of hyperglycaemia (≥ 10 mmol/L)			Admissions had at least one episode of significant hyperglycaemia (≥ 14 mmol/L)			Total
		No	Yes	P-value	No	Yes	P-value	No	Yes	P- value	N=4,901
		N=3,598	N=360		N=1,505	N=2,453		N=2,620	N=1,338		
Age at admission (years) [mean ± SD]		66.7 (15.5)	64.6 (16.0)	0.01	69.1 (14.0)	64.9 (16.3)	<0.01	68.2 (14.4)	63.4 (17.2)	<0.01	67.0 (15.4)
	15-39	227 (6.3%)	33 (9.2%)	0.07	46 (3.1%)	214 (8.7%)	<0.01	106 (4.0%)	154 (11.5%)	<0.01	292 (6.0%)
Age group	40-65	1,269 (35.3%)	132 (36.7%)		504 (33.5%)	897 (36.6%)		900 (34.4%)	501 (37.4%)		1,695 (34.6%)
	65+	2,102 (58.4%)	195 (54.2%)		955 (63.5%)	1,342 (54.7%)		1,614 (61.6%)	683 (51.0%)		2,914 (59.5%)
Gender	Female	1,551 (43.1%)	180 (50.0%)	0.01	673 (44.7%)	1,058 (43.1%)	0.33	1,136 (43.4%)	595 (44.5%)	0.51	2,168 (44.2%)
	Male	2,047 (56.9%)	180 (50.0%)		832 (55.3%)	1,395 (56.9%)		1,484 (56.6%)	743 (55.5%)		2,733 (55.8%)
Māori	Non-Māori	2,357 (65.9%)	211 (59.1%)	<0.01	948 (63.4%)	1,620 (66.5%)	0.05	1,672 (64.3%)	896 (67.3%)	0.06	3,228 (66.4%)
	Māori	1,218 (34.1%)	146 (40.9%)		547 (36.6%)	817 (33.5%)		929 (35.7%)	435 (32.7%)		1,632 (33.6%)
Type of diabetes	Type 1 Type 2	273 (7.6%) 3,325 (92.4%)	67 (18.6%) 293 (81.4%)	<0.01	51 (3.4%) 1,454 (96.6%)	289 (11.8%) 2,164 (88.2%)	<0.01	118 (4.5%) 2,502 (95.5%)	222 (16.6%) 1,116 (83.4%)	<0.01	373 (7.6%) 4,528 (92.4%)
30 days readmission		560 (15.6%)	53 (14.7%)	0.67	225 (15.0%)	388 (15.8%)	0.46	398 (15.2%)	215 (16.1%)	0.47	731 (14.9%)
60 days readmission		740 (20.6%)	91 (25.3%)	0.04	303 (20.1%)	528 (21.5%)	0.30	533 (20.3%)	298 (22.3%)	0.16	996 (20.3%)
90 days readmission		861 (23.9%)	105 (29.2%)	0.03	342 (22.7%)	624 (25.4%)	0.05	606 (23.1%)	360 (26.9%)	<0.01	1,156 (23.6%)
365 days readmission		1,152 (32.0%)	141 (39.2%)	<0.01	455 (30.2%)	838 (34.2%)	0.01	808 (30.8%)	485 (36.2%)	<0.01	1,521 (31.0%)
Length of stay (weeks)											
[mean ± SD]		1.3 (1.8)	2.2 (2.8)	<0.01	1.1 (1.5)	1.6 (2.2)	<0.01	1.2 (1.7)	1.7 (2.3)	<0.01	1.3 (1.8)
Admission to HDU/ICU		13 (0.4%)	1 (0.3%)	0.80	5 (0.3%)	9 (0.4%)	0.86	11 (0.4%)	3 (0.2%)	0.33	19 (0.4%)
	Target Range	2,300 (63.9%)	291 (80.8%)	<0.01	1,490 (99.0%)	1,101 (44.9%)	<0.01	2,274 (86.8%)	317 (23.7%)	<0.01	2,591 (52.9%)
	Mildly										
	Hyperglycaemia	968 (26.9%)	45 (12.5%)		0 (0.0%)	1,013 (41.3%)		331 (12.6%)	682 (51.0%)		1,013 (20.7%)
Glucose Category	Significant Hyperglycaemia	330 (9.2%)	9 (2.5%)		0 (0.0%)	339 (13.8%)		0 (0.0%)	339 (25.3%)		339 (6.9%)
	Hypoglycaemia	0 (0.0%)	15 (4.2%)		15 (1.0%)	0 (0.0%)		15 (0.6%)	0 (0.0%)		15 (0.3%)
	Missing	0 (0.0%)	0 (0.0%)		0 (0.0%)	0 (0.0%)		0 (0.0%)	0 (0.0%)		943 (19.2%)
Mean blood glucose [mean ± SD]		9.5 (3.2)	7.8 (2.5)	<0.01	6.8 (1.3)	11.0 (3.0)	<0.01	7.8 (1.9)	12.4 (3.1)	<0.01	9.4 (3.2)
Glucose checked at least once every 24 hours		1,073 (29.8%)	203 (56.4%)	<0.01	280 (18.6%)	996 (40.6%)	<0.01	650 (24.8%)	626 (46.8%)	<0.01	1,276 (26.0%)
Ketones checked at least once during admission in Type 1 diabetes patients		90 (33.0%)	21 (31.3%)	0.80	6 (11.8%)	105 (36.3%)	<0.01	23 (19.5%)	88 (39.6%)	<0.01	113 (30.3%)
BGL checks per 24 hours of stay	<1 BGL check 1-3 BGL checks ≥4 BGL checks Missing	2,525 (70.2%) 1,044 (29.0%) 29 (0.8%) 0 (0.0%)	157 (43.6%) 194 (53.9%) 9 (2.5%) 0 (0.0%)	<0.01	1,225 (81.4%) 279 (18.5%) 1 (0.1%) 0 (0.0%)	1,457 (59.4%) 959 (39.1%) 37 (1.5%) 0 (0.0%)	<0.01	1,970 (75.2%) 594 (44.4%) 32 (2.4%) 0 (0.0%)	712 (53.2%) 594 (44.4%) 32 (2.4%) 0 (0.0%)	<0.01	2,682 (54.7% 1,238 (25.3%) 38 (0.8%) 943 (19.2%)
HbA1c checked within 3 months of admission		2,187 (60.8%)	234 (65.0%)	0.12	852 (56.6%)	1,569 (64.0%)	<0.01	1,556 (59.4%)	865 (64.6%)	<0.01	2,932 (59.8%)
HbA1c values [mean ± SD]		63.2 (22.2)	67.4 (23.1)	<0.01	52.7 (15.4)	69.6 (23.2)	<0.01	57.2 (18.3)	75.1 (24.2)	<0.01	61.9 (21.7)

**Table 2.** Monitoring of glucose levels and glycaemic control and outcomes

Variables	Adjusted odds ratios from logistic regressions						OLS regression
	Mortality within 1 year	Mortality within 60 days	30 days readmission	60 days readmission	90 days readmission	365 days readmission	Length of stay (weeks)
Hypoglycaemia							
No	1	1	1	1	1	1	1
Yes	1.94***	1.90***	0.91	1.24	1.22	1.24	3.13***
	(1.51 - 2.49)	(1.37 - 2.64)	(0.67 - 1.24)	(0.96 - 1.61)	(0.95 - 1.56)	(0.99 - 1.56)	(2.55 - 3.85)
Any hyperglycaemia							
No	1	1	1	1	1	1	1
Yes	1.02	1.07	1.07	1.04	1.06	1.05	1.56***
	(0.84 - 1.23)	(0.82 - 1.40)	(0.86 - 1.33)	(0.85 - 1.26)	(0.88 - 1.27)	(0.89 - 1.25)	(1.35 - 1.80)
Significant hyperglycaemia							
No	1	1	1	1	1	1	1
Yes	1.37**	1.26	1.06	1.10	1.16	1.20*	1.54***
	(1.12 - 1.66)	(0.96 - 1.66)	(0.85 - 1.32)	(0.90 - 1.34)	(0.97 - 1.40)	(1.01 - 1.42)	(1.32 - 1.79)
Glucose checks at least once per 24 hours							
No	1	1	1	1	1	1	1
Yes	0.77**	0.83	1.08	1.11	1.11	1.20*	0.36***
	(0.64 - 0.91)	(0.64 - 1.07)	(0.89 - 1.32)	(0.93 - 1.32)	(0.94 - 1.31)	(1.03 - 1.40)	(0.31 - 0.41)
Type of diabetes							
Type 1	1	1	1	1	1	1	1
Type 2	1.14	2.77*	1.09	1.04	0.92	0.98	1.37*
	(0.78 - 1.67)	(1.27 - 6.04)	(0.74 - 1.59)	(0.75 - 1.45)	(0.68 - 1.25)	(0.74 - 1.30)	(1.07 - 1.76)
Ethnicity							
Non-Māori	1	1	1	1	1	1	1
Māori	1.37***	1.12	1.27*	1.44***	1.39***	1.37***	0.88
	(1.15 - 1.62)	(0.88 - 1.42)	(1.05 - 1.53)	(1.22 - 1.70)	(1.19 - 1.63)	(1.18 - 1.59)	(0.78 - 1.00)
Age							
40-65	1	1	1	1	1	1	1
15-39	0.30***	.	0.97	0.94	1.01	1.05	0.92
	(0.17 - 0.54)	.	(0.64 - 1.48)	(0.65 - 1.36)	(0.72 - 1.42)	(0.76 - 1.44)	(0.70 - 1.22)
65+	2.27***	1.84***	1.10	1.09	1.09	1.08	1.28***
	(1.90 - 2.72)	(1.43 - 2.37)	(0.91 - 1.34)	(0.91 - 1.29)	(0.93 - 1.29)	(0.93 - 1.26)	(1.12 - 1.46)
Constant	0.13***	0.02***	0.14***	0.19***	0.26***	0.35***	2.26***
	(0.09 - 0.20)	(0.01 - 0.05)	(0.09 - 0.21)	(0.13 - 0.28)	(0.18 - 0.36)	(0.26 - 0.49)	(1.72 - 2.98)

Notes: *** p<0.001, ** p<0.01, * p<0.05. 95% confidence intervals are in parentheses

## 3 Results

Over the selected time period a total cohort of 4,901 admissions in 3,380 diabetes patients was assessed, of which 80.8% (n=4901) had their blood glucose levels monitored (including 51,680 blood glucose measurements). Individual patients were admitted a mean of 1.6 (+/-1.1) times during the 16-month study period, and the demographic and clinical characteristics of this group are shown in Table 1.

The mean age of participants was 67 years and admitted patients with at least one episode of hypoglycaemia, hyperglycaemia or significant hyperglycaemia tended to be younger (64.6 vs 66.7 years, 64.9 vs 69.1 years, and 63.4 vs 68.2 years respectively). The mean length of stay was 1.3 weeks. Approximately 9.5% of admitted patients died within 60 days of admission and 22.6% of admitted patients died within one year. Overall, the mean number of glucose measurements taken over a 24-hour period was 0.7 (+/-0.8). One quarter (26%; n=1276) of admissions had one or more glucose level recorded for each 24-hour period of their hospital stay, though only 0.8% of admissions had blood glucose levels recorded at least four times (Table 1).

**Figure 1.**
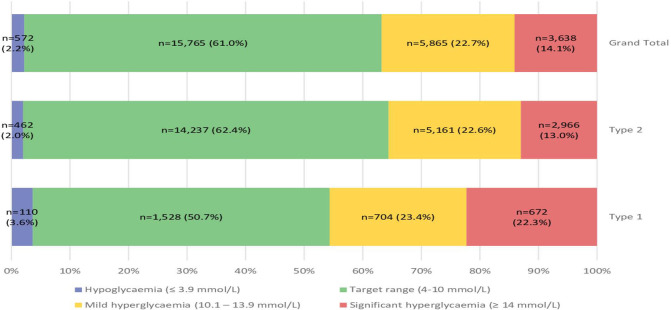
All glucose readings in the study by diabetes ICD coding type

Overall, 61% of blood glucose measurements were in target range (Figure 1). A total of 45.7% (n=1376) of measurements were above target range in those with type 1 diabetes, compared to only 35.6% (n=8127) in those with type 2 diabetes group (P < 0.001). Similarly, hypoglycaemia was nearly twice as likely in those with type 1 diabetes (3.6% of measurements compared to 2.0% in those with type 2 diabetes; P < 0.001; Figure 1). No recorded capillary ketone assessment was performed at any point during the hospital stay of 30.3% (n=133) type 1 diabetes admissions, and only 1.9% (n=7) patients with type 1 diabetes had one or more level checked on each day of admission. HbA1c had been performed during or within the preceding three months in 59.8% of admissions; the mean HbA1c was 61.9 (+/-21.7) mmol/mol.

There was a statistically significant association noted between hypoglycaemia and mortality as well as length of stay (Table 2). Approximately 33.3% of admitted patients with hypoglycaemia died within one year following admission while only 22% without hypoglycaemia died within one year. After adjusting for type of diabetes, ethnicity, age, any hyperglycaemia, significant hyperglycaemia, and glucose checks, patients experiencing at least one episode of hypoglycaemia were 1.90 times (CI: 1.37-2.64) and 1.94 times (CI: 1.51-2.49) more likely to die within 60 days and one year respectively following admission (Table 2). In addition, admitted participants with hypoglycaemia had an increased length of hospital stay by a mean of 3.13 weeks (CI: 2.55-3.85). There was no association found between inpatient hypoglycaemia and hospital readmission. Patients with any episode of hyperglycaemia had an increased length of stay by a mean of 1.56 weeks (CI: 1.35-1.80). After adjustment, the association between hyperglycaemia and both mortality and readmission were insignificant. However, admitted participants with at least one episode of significant hyperglycaemia had a 37% greater one-year mortality, 20% higher risk of readmission within one year and extended hospital stays (1.54 weeks), all of which were statistically significant. Importantly, hospitalised patients who had their blood glucose tested at least once every 24 hours were less likely to die within one year after admission (Adjusted odds ratios (OR): 0.77, CI: 0.64-0.91) and less likely to have prolonged hospital stays (0.36 weeks) than those who did not. There was no relationship found between socioeconomic status and dysglycaemia.

Māori patients accounted for 33.6% of total admissions. Māori were statistically significantly more likely than non-Māori to experience at least one episode of hypoglycaemia (OR: 1.46, 95% CI: 1.16-1.85) and less likely to experience hyperglycaemia (OR: 0.80, CI: 0.69-0.93) or significant hyperglycaemia (OR: 0.80, CI 0.69-0.93) after adjusting for age and type of diabetes. Māori patients with diabetes also had a statistically significant higher one-year mortality as well as higher readmission rates at 30, 60, 90 and 365 days than nonMāori patients (Table 2).

## 4 Discussion

The frequency of inpatient glycaemic monitoring at our tertiary centre is clearly suboptimal and far from meeting the recommended standards. Furthermore, a substantial number of the glucose levels recorded fall outside the recommended target range for in patients with diabetes. At the time of the study, Waikato hospital did not have a standardised tool to explicitly order point of care glucose and ketone testing leaving some ambiguity to the monitoring and frequency of testing. This reason along with clinical inertia may go some way to explaining why 19.2% of the admission cohort did not have any glucose monitoring, which is concerningly high and puts patients at risk of late detection of hypoglycaemia, hyperosmolar hyperglycaemic state and diabetic ketoacidosis. Other explanations postulated for this include lack of awareness of medical and nursing staff of the importance of frequent glycaemic monitoring, heavy nursing workloads as well as patients and nursing staff relying on the patient’s own glucometers while in hospital. A tool has subsequently been implemented at Waikato hospital allowing standardisation of inpatient diabetes care which should ameliorate the problem of poor glycaemic monitoring and control.

Hyperglycaemia intensifies the body’s pro-inflammatory responses, potentiates oxidative injury and is pro-thrombotic thereby contributing to poorer outcomes in life threatening conditions including acute myocardial infarction, stroke and congestive cardiac failure [12]. There is also a higher incidence of postoperative infection and surgical would complications with hyperglycaemia [13]. It is therefore perhaps not surprising that inpatients with suboptimal control had a higher one-year mortality following discharge than those with target range blood glucose levels.

We speculate reasons that Māori patients had more hypoglycaemia than non-Māori patients may include overly aggressive treatment of abnormal glucose measurements in hospital as well as recurrent covert non-adherence to diabetes treatment in the community leading to poorer glycaemic control resulting in repeated up-titration of insulin and diabetes medications, with subsequent hypoglycaemia when all these medications are given at once in hospital. Māori have substandard health outcomes and an increased risk of complications from diabetes in New Zealand [14]. This study affirms that Māori with diabetes have higher hospital readmission rates and higher one-year mortality post discharge than non-Māori, although it remains unclear how significant the glycaemic abnormalities in those with diabetes demonstrated in this study contribute to this [15]. Due to the paucity of data on glycaemic control in this study, further studies are required to access whether diabetes was the primary cause of these outcomes. Further diabetes primary prevention strategies and significant changes in the framework of how we delivery health services to Māori are required. Treatment plans involving both the patient and their whanau are essential for preventing and treating diabetes in Māori [16].

Further, approximately two fifths of patients with diabetes did not have a HbA1c measurement from the preceding three months. The reasons for this are likely multifactorial including clinical inertia, measurement out of area, social deprivation as well as poor patient access to general practice and laboratory sites. Nevertheless, a hospital admission should have provided the opportunity for HbA1c testing with remediable action taken if suboptimal glycaemic control was identified.

It is recommended that type 1 diabetes patients have a blood ketone level checked if the blood glucose is persistently over 15 mmol/L or if unwell [17]. Checking ketones when patients are unwell, irrespective of blood glucose levels is of particular importance of late with the increase in eugylcaemic ketoacidosis associated with sodium-glucose co-transporter 2 inhibitors [18]. At the time of the study these drugs were not available in New Zealand therefore ketones were not assessed in the type 2 diabetes patients. It is concerning that despite a high proportion of patients with type 1 diabetes having significant hyperglycaemia, only 30.3% (n=133) of admissions had their ketones checked during their admission. Although unlikely, it is possible that medical teams were reliant on beta-hydroxybutyrate levels from the lab, particularly in patients with an increased likelihood of diabetic ketoacidosis, but this data was not included in this study. A minority of patients may also have been receiving IV insulin at the time with resolving hyperglycaemia and therefore ketones may not have been checked for every value out of range when glucose levels were falling.

There were several limitations identified in the study. Insulin and diabetes medication use in individual patients was not explored. Venous blood gas (VBG) analyses were not included in the study. VBG testing is an essential component of a diabetic ketoacidosis diagnosis and monitoring plan as well as being frequently used by intensive care units. However, there were only a small number of ICU and HDU patients involved in the study suggesting that inclusion of VBG glucose levels would not have significantly altered our results. Only electronic data was used but the laboratory ensured all point of care testing data was regularly uploaded from every ward during the study period. Those admitted for less than 24 hours were excluded from the study, but we hypothesise that rates of glucose and ketone monitoring may have been lower in this group due to milder illness.

In the era of rapidly advancing diabetes technologies, it is more commonplace for diabetes patients to be utilising continuous glucose monitoring and flash glucose monitoring. Whilst these systems have been shown to improve glycaemic control [19], they have up to recently not been approved for inpatient glucose monitoring use, especially given the lag time between blood and interstitial glucose concentrations with some of these devices [20]. There is also a theoretical increased risk of reduced functionality following exposure to radiological equipment with these devices. However, recent studies have shown that they retain basic functionality and data storage under such conditions [21]. As continuous glucose monitoring technology improves and lag time and accuracy of readings approach real time blood glucose readings, approving their use as valid tools to measure inpatient blood glucose levels is an effective way of increasing glycaemic monitoring in diabetes inpatients. Indeed, inpatient use of real-time continuous glucose monitoring using a glucose telemetry system has already been shown to reduce hypoglycaemia in high risk type 2 diabetes patients on insulin [22]. These systems would also help to reduce workloads for frequently busy and under-staffed nursing departments as well as increasing patient satisfaction by reducing the pain and trauma relating to multiple daily finger prick glucose tests.

This study demonstrates the need to prioritise glycaemic monitoring and control of inpatients with diabetes in order to enable more rapid recovery from acute illness, reduce morbidity, and decrease the length of hospital stays and costs. Previous studies have demonstrated that healthcare workers including nursing staff, pharmacists and junior doctors have low confidence when managing diabetes [23]. With the ever-increasing numbers of diabetes patients in hospital a large proportion of their care is being provided by non-specialist staff. Face to face or online diabetes education directed towards hospital healthcare providers is therefore essential; with online healthcare professional education recently being shown to be an effective strategy in improving glucose control in these patients [24].

Strategies focusing on the system of diabetes management as well as patient-mediated quality improvement factors are vital [25]. Creating clear and easily accessible diabetes clinical practice guidelines that are user friendly would help solve the issues of time efficiency and therapeutic inertia that have been identified as reasons for non-adherence to diabetes hospital protocols in the past [26]. Multidisciplinary diabetes teams are also fundamental in helping to improve acute and chronic care of such patients as well as facilitating self-management both in the hospital setting and the community [27]. This holistic approach towards diabetes care can augment patient satisfaction and empower patients to become more independent and achieve their glycaemic goals. Increasing the size of such teams as well as employing more hospital-based diabetes nurses would help to optimise patient centred care and the diabetes education of healthcare staff.

In conclusion, both significant hyperglycaemia and hypoglycaemia were associated with increased morbidity as well as longer hospital stays emphasising the importance of maintaining good glycaemic monitoring and control in hospitalised diabetes patients. Inpatient hypoglycaemia was associated with increased mortality rates and was more common in Māori patients with diabetes. At the time of the study, both monitoring and glycaemic control were suboptimal, with strategies required to improve this. However, given the ever-growing use of diabetes technologies and resources available to aid in diabetes management, a significant improvement in these results is expected in the near future.

## Abbreviations

GDP: Gross domestic product

HDU: High dependency unit

ICU: Intensive care unit

VBG: Venous blood gas

HbA1c: Glycosylated hemoglobin
